# Learning From a Missed Diagnosis: Complete Agenesis of the Corpus Callosum With Benign Hydrocephalus

**DOI:** 10.1002/ccr3.70693

**Published:** 2025-07-29

**Authors:** J. E. Samaranayake, S. M. Kankanamge, P. Pirakash, U. A. Liyanage, Y. Mathangasinghe, D. G. Gonsalvez, N. Gunasekera

**Affiliations:** ^1^ Postgraduate Institute of Medicine University of Colombo Colombo Sri Lanka; ^2^ Faculty of Medicine University of Colombo Colombo Sri Lanka; ^3^ Centre for Human Anatomy Education, Department of Anatomy and Developmental Biology, Biomedical Discovery Institute Monash University Melbourne Victoria Australia; ^4^ Department of Anatomy and Developmental Biology, Biomedical Discovery Institute Monash University Melbourne Victoria Australia; ^5^ Department of Neurosurgery Teaching Hospital Karapitiya Galle Sri Lanka

**Keywords:** arrested hydrocephalus, benign hydrocephalus, corpus callosum, corpus callosum agenesis

## Abstract

In agenesis of the corpus callosum, ventriculomegaly without evidence of cerebrospinal fluid obstruction often requires no surgical intervention. Regular follow‐up every 3–6 months, then annually, is essential to monitor for signs of raised intracranial pressure or neurological decline—prompting advanced imaging and consideration of surgical management if deterioration occurs.

## 1 Introduction

**FIGURE 1:**
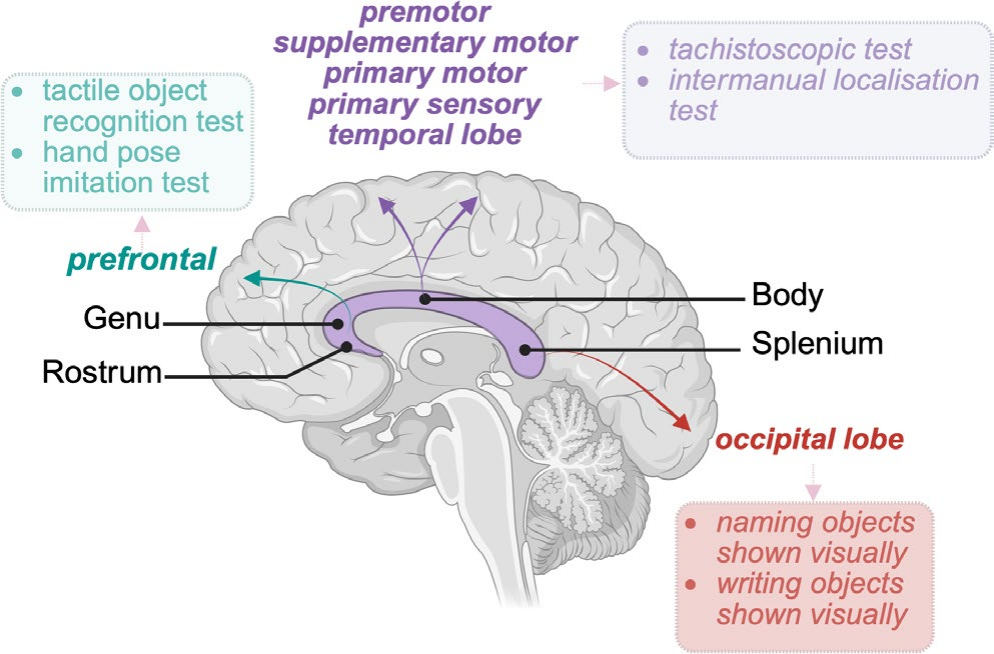
The parts of the corpus callosum and their complex connections with specific brain regions. Clinical tests used to assess intricate neurocognitive functions related to specific parts of the corpus callosum are listed in the boxes.

The corpus callosum is the primary commissural white matter tract connecting the two cerebral hemispheres. It comprises the rostrum, genu, body, and splenium, forming complex interhemispheric connections that support intricate neurocognitive functions (Figure 1). Agenesis of corpus callosum (AgCC), first described in 1812 by Reil [1], is a rare congenital anomaly [2] with a prevalence of approximately 3–5 per 10,000 births [3]. It is classified into three subtypes: partial agenesis (absence of a segment), complete agenesis (absence of the entire corpus callosum), and hypoplasia (uniform underdevelopment) [4]. AgCC is frequently associated with additional anomalies involving the cardiac, gastrointestinal, genitourinary, vascular, or central nervous systems [3], collectively termed syndromic AgCC. The most common central nervous system malformations include cerebellar and brainstem abnormalities [3]. Isolated AgCC, especially in the presence of genetic abnormalities, is considerably rarer.

Patients with AgCC present with a broad spectrum of neuropsychiatric and cognitive symptoms, ranging from asymptomatic individuals with near‐normal cognitive functions to those with profound intellectual disabilities and intractable seizures [5, 6, 7]. AgCC can be classified into two main types: Type 1, in which axons form but fail to cross the midline (leading to Probst bundle formation), and Type 2, where commissural axons fail to form entirely, resulting in the absence of Probst bundles. Most cases are diagnosed antenatally or in early childhood, with nearly all identified by adolescence when increased cognitive demands reveal underlying deficits [6, 8]. In rare instances, congenital benign ventriculomegaly is associated with AgCC [9]. We report an unusual case of isolated Type 2 AgCC with communicating ventriculomegaly affecting the posterior horns—diagnosed only at age 18. This case underscores the importance of non‐surgical management of benign causes of hydrocephalus to avoid potential complications such as intracranial hemorrhage.

## 2 Case Examination

An 18‐year‐old Sri Lankan man (assigned male at birth) presented to the Emergency Treatment Unit, Teaching Hospital Karapitiya with a sudden‐onset, diffuse headache (9/10 on the Likert pain scale), low‐grade fever, and upper respiratory symptoms (nasal congestion and rhinitis) for 2 days. He reported no history of seizures, chronic headaches, loss of consciousness, or head trauma, and denied any visual or auditory impairments. He was the third child of non‐consanguineous parents, born at term following an uncomplicated pregnancy and delivery. Antenatal and neonatal periods were unremarkable. Developmental milestones were normal across all domains, except for a slight delay in forming complete sentences, resolved by 2.5 years with speech therapy. No neurocognitive or psychiatric concerns were noted during the school years. Family history revealed no congenital anomalies over three generations. The mother had no miscarriages or children with congenital malformations and denied exposure to teratogens, alcohol, or congenital infections during pregnancy. Physical and neurological examinations were unremarkable, with no signs of papilledema, visual field defects, meningism, or gait disturbances.

## 3 Methods

**FIGURE 2:**
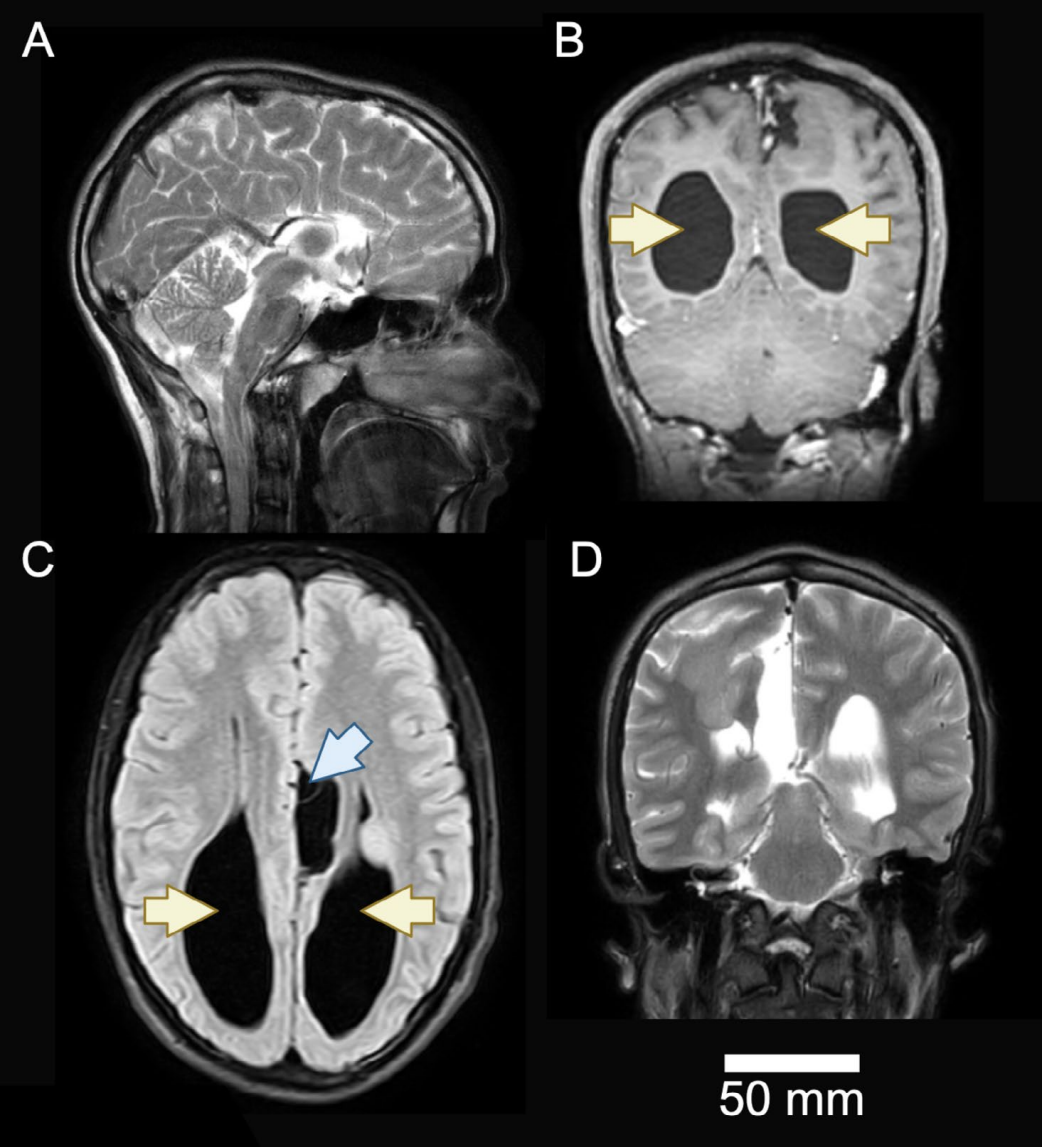
MRI images of the patient with agenesis of corpus callosum. (A) T2‐weighted sagittal MRI, (B) T1‐weighted coronal MRI, (C) FLAIR axial, (D) T2‐weighted coronal MRI (at the level of anterior horns). Yellow arrows indicate enlarged lateral ventricles, while the blue arrow points to an interhemispheric cyst.

Blood investigations performed at the time of initial presentation included a white blood cell count of 10.38 × 10^3^/mm^3^ (reference range: 4.5–11 × 10^3^/mm^3^), C‐reactive protein of 5 mg/dL (≤ 5 mg/dL), serum sodium of 135 mmol/L (135–145 mmol/L), serum potassium of 3.9 mmol/L (3.5–5.5 mmol/L), and a negative blood culture. The severity and persistence of the headache warranted a computed tomography (CT) scan, which showed ventriculomegaly. Subsequently, magnetic resonance imaging (MRI) was performed to exclude sinister pathologies such as brain tumors. T1W, T2W, DWI, and FLAIR sequences with post‐contrast imaging showed gross dilation of the occipital horns (colpocephaly), normal remaining ventricles, complete AgCC, everted cingulate gyri, crescent‐shaped frontal horns, and cerebral oedema (Figure 2). An interhemispheric cyst was seen in the left parasagittal area, with heterotopic gray matter near the subependymal region of the lateral ventricle. No mass lesions or additional malformations were found. Diffusion tensor imaging revealed absence of compensatory white matter tracts.

Following diagnosis of AgCC, developmental delay was assessed using the Montreal Cognitive Assessment (MoCA). The patient scored 17 out of 30 on the MoCA [visuospatial domain: 4/5; naming: 3/3; attention: 3/6; language: 0/3; abstraction: 1/2; delayed recall: 2/5; orientation: 4/6]. Tests for split‐brain function, including tachistoscopic tests, dual‐response tasks, and lateralizing language function assessments, revealed no abnormalities. Additionally, no craniofacial dysmorphism, skeletal anomalies, dermatological findings, genital abnormalities, or limb deformities were observed.

The patient improved the next day with conservative management (paracetamol, steam inhalation, nasal drops). Virological studies were not performed due to resource constraints. He remained under observation for signs of raised intracranial pressure, which did not develop. No neurological deterioration was noted.

## 4 Results

A working diagnosis of benign ventriculomegaly with viral upper respiratory tract infection was made. The patient was managed symptomatically for upper respiratory tract infection and conservatively for colpocephaly. At 6‐month follow‐up, he remained asymptomatic—no headaches, seizures, or cognitive decline—indicating a favorable outcome with non‐surgical management.

## 5 Discussion

The pathogenesis of AgCC remains largely unclear. About 70% of cases have no identifiable cause, with foetal alcohol syndrome being the most common acquired etiology [10, 11]. Other associated risk factors include perinatal infections, chromosomal anomalies, multiple gestations, advanced parental age, prematurity, male sex, and certain ethnicities [3, 6].

In this case, hydrocephalus may have resulted from progressive childhood ventriculomegaly that spontaneously arrested, as evidenced by normal head circumference. The exact timing and mechanism of long‐standing overt ventriculomegaly in adults (LOVA) remain poorly understood [12].

Misinterpreting benign hydrocephalus as pathological may lead to unnecessary interventions; hence, a thorough understanding of this anatomical variant is essential. AgCC is typically diagnosed during prenatal ultrasonography, confirmed via fetal MRI or detailed ultrasound [6]. This evaluation may include amniocentesis to screen for genetic defects and congenital infections [2]. A subset of cases is diagnosed during early childhood due to developmental abnormalities, but it is exceedingly rare to identify AgCC in adulthood, as in this case. Most patients exhibit neurocognitive and behavioral deficits during neuropsychological evaluations [8]. Although some reports claim “normal” cognitive function in individuals with AgCC [6], it is crucial to note that commonly used tools like the Mini‐Mental State Examination (MMSE) lack the sensitivity to detect mild cognitive impairments, necessitating comprehensive neuropsychological assessments (reviewed in [6]). Patients with AgCC may display intact word‐generation abilities but often struggle with linguistic pragmatics, including understanding idioms, proverbs, rhymes, humor, syntax, and figurative language [13]. Even in the absence of overt symptoms, subtle developmental delays—as in this case—justify detailed evaluation and follow‐up. However, not all such delays warrant immediate advanced imaging.

Interestingly, split‐brain functional deficits are not common in AgCC cases and depend on the extent of agenesis and compensation by other commissures. The brain's remarkable capacity for neuroplasticity enables the development of compensatory structures like Probst bundles and heterotopic bundles in some patients with AgCC [4]. The absence of split‐brain signs may reflect unique developmental trajectories, with increased local but reduced global brain connectivity [14].

**FIGURE 3:**
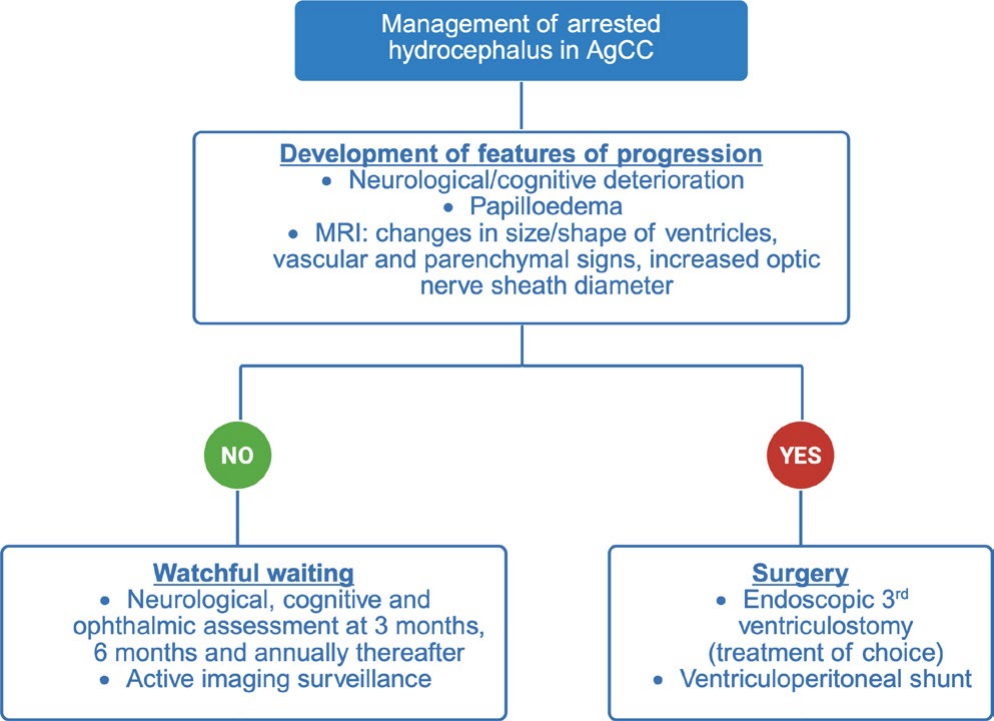
Management protocol for agenesis of corpus callosum (AgCC), developed based on current published evidence in the absence of universal guidelines. Following the diagnosis of AgCC, if there are no signs of raised intracranial pressure or clinical deterioration, patients may be monitored with neurocognitive assessments conducted initially every 3 months, then every 6 months, and subsequently on an annual basis. Any clinical decline should prompt advanced neuroimaging before considering surgical intervention.

There is no consensus on managing LOVA associated with complete AgCC. Evidence supports conservative management in patients with arrested hydrocephalus with no worsening neurocognitive symptoms or radiological deterioration on serial imaging [15, 16]. Shunting procedures in arrested hydrocephalus carry a significant risk of complications, such as subdural hematomas due to over‐drainage [17]. We propose a management protocol for arrested AgCC, based on current evidence (Figure 3). While conservative management shows promise, long‐term stability is not guaranteed; some cases may eventually require surgery [15, 17]. Thus, regular neuropsychological assessments and periodic imaging remain essential to detect progression and guide intervention. This case illustrates the diagnostic and therapeutic complexity of isolated AgCC presenting in adulthood with benign ventriculomegaly. Early recognition and neurocognitive evaluation can reveal subtle deficits that may otherwise be overlooked. Conservative management with biannual follow‐up appears to be effective and safe, particularly in resource‐limited settings. Advanced imaging should be reserved for cases showing neurological deterioration during 3–6 monthly or annual follow‐ups to balance clinical benefit with feasibility.

## Author Contributions

**J. E. Samaranayake:** data curation, investigation, project administration, writing – original draft, writing – review and editing. **S. M. Kankanamge:** investigation. **P. Pirakash:** investigation, resources. **U. A. Liyanage:** methodology, supervision, writing – review and editing. **D. G. Gonsalvez:** investigation, methodology, supervision, writing – review and editing. **Y. Mathangasinghe:** methodology, writing – original draft, writing – review and editing. **N. Gunasekera:** methodology, project administration, writing – original draft, writing – review and editing.

## Ethics Statement

The authors have nothing to report.

## Consent

We obtained written informed consent from the patient to publish this case report in accordance with the journal's patient consent policy.

## Conflicts of Interest

The authors declare no conflicts of interest.

## Data Availability

The original contributions presented in the case report are included in the article; further inquiries can be directly made to the corresponding author.
